# From Plate to Mind: Scientific Perspectives on Foods That May Influence Anxiety and Depression

**DOI:** 10.3390/nu18091318

**Published:** 2026-04-22

**Authors:** Antoniya Hachmeriyan, Gabriela Panayotova, Hristiyana Todorova

**Affiliations:** Division of Physiology, Department of Physiology and Pathophysiology, Medical University of Varna, 9002 Varna, Bulgaria; gabrielapanayotova95@gmail.com (G.P.); or hristiyana.todorova@mu-varna.bg (H.T.)

**Keywords:** nutritional psychiatry, omega-3, EPA, DHA, microbiome, gut–brain axis, inflammation, vitamins, minerals, depression, anxiety

## Abstract

**Background:** Nutritional psychiatry increasingly links diet quality and specific bioactive nutrients to depression and anxiety outcomes. Mechanistic evidence implicates neuroimmune activation, inflammation, altered neurotransmitter synthesis, and microbiota-derived metabolites. **Objective:** The objective of this study is to synthesize evidence on omega-3 polyunsaturated fatty acids (*n*-3 PUFAs), the microbiota–gut–brain axis, and vitamins and minerals that influence neurotransmitter synthesis, inflammation, and brain function and to translate these findings into food-based strategies. **Methods:** This study consisted of a focused synthesis of randomized controlled trials (RCTs), meta-analyses, and systematic reviews indexed in PubMed, Scopus and Web of Science, selected for relevance to omega-3s, probiotics/prebiotics, dietary patterns, and micronutrients (folate/B-vitamins, vitamin D, magnesium, zinc, and vitamin C/copper pathways). **Results:** RCT and meta-analytic evidence suggest modest benefits of omega-3 supplementation for anxiety severity and depressive symptoms, with heterogeneity by dose, EPA: DHA composition, and baseline inflammatory status. The gut–brain axis literature supports bidirectional effects of stress and microbiota, and meta-analyses of probiotics/prebiotics show small improvements in depressive and anxiety symptoms, likely dependent on strain and host phenotype. Micronutrients serve as enzymatic cofactors for monoamine and GABA synthesis and modulate immune signaling; clinical effects are the most consistent when correcting insufficiency or in biomarker-defined subgroups. A whole-diet RCT demonstrates that structured dietary improvement can reduce depressive symptoms as adjunctive therapy. **Conclusions:** A food-first approach emphasizing Mediterranean-style dietary patterns, omega-3-rich seafood, a diverse array of fiber, and micronutrient density is the most defensible. Supplementation may be considered selectively, guided by clinical context and nutritional status.

## 1. Introduction

Depression and anxiety disorders are leading contributors to global disability. They are increasingly recognized as heterogeneous syndromes with overlapping biological pathways [1]. Beyond monoamine signaling, contemporary frameworks for depression and anxiety highlight neuroimmune activation, oxidative stress, metabolic dysregulation, altered neuroplasticity, and disturbed stress physiology. Diet is a plausible modulator of these systems because it simultaneously shapes cell signaling, micronutrient cofactor availability, and the intestinal microbiome. Accordingly, nutritional psychiatry has progressed from observational associations to controlled trials testing dietary change and targeted supplementation [2].

Several mechanisms are proposed to contribute to the onset and progression of mood disorders.

### 1.1. Mood Disorders May Be the Manifestation of Inborn Errors of Metabolism

Mood disorders are usually approached as primary psychiatric illnesses. Yet a clinically important subset reflects inborn errors of metabolism (IEMs)—genetic defects in enzymes or transporters that disrupt key biochemical pathways in the brain and body. In pediatric and young adult populations, psychiatric symptoms can precede overt neurologic signs, and delayed recognition may allow for preventable or irreversible CNS injury [3].

Across the IEM spectrum, mood pathology is not rare. A comprehensive review of psychiatric presentations reported that psychiatric symptoms span attention problems, anxiety, mood/behavioral disorders, and psychosis and identified > 100 IEMs associated with psychiatric manifestations; in a curated analysis, 94 IEMs were linked to psychiatric symptoms, with mood changes ranging from depressive syndromes to bipolar-like presentations [3]. Similarly, a systematic review focusing on adolescents and adults emphasized that late-onset IEMs may appear with predominantly psychiatric symptoms, complicating differentiation from primary mood disorders [4].

From a pathophysiological standpoint, IEMs can drive mood symptoms through several converging routes: (1) toxic accumulation of metabolites (e.g., ammonia, porphyrin precursors, copper) that alter neuronal function; (2) energy failure due to mitochondrial dysfunction; and (3) disruption of excitatory–inhibitory balance and monoaminergic neurotransmission, which can manifest as depression, mood lability, irritability, or manic episodes [5]. In the pediatric population, mood and anxiety disorders are among the most frequent psychiatric phenotypes of IEMs, including symptoms such as insomnia, anergia, apathy, social withdrawal, appetite changes, mood instability, and manic states [3,6].

Clinically, mood disorders associated with IEMs often exhibit characteristic diagnostic patterns that may aid early recognition. Acute psychiatric episodes may mimic delirium, presenting with confusion, disorganized thinking, and incoherent speech. In such cases, particularly when psychiatric symptoms occur in the context of acute encephalopathy, measurement of plasma ammonia is essential, as hyperammonemia caused by urea cycle disorders represents a potentially treatable metabolic emergency [6,7].

Certain metabolic conditions may manifest primarily with psychiatric symptoms and therefore remain underdiagnosed. Acute intermittent porphyria and related hepatic porphyrias are well-known examples, frequently presenting with depression, anxiety, hallucinations, or psychosis. These neuropsychiatric manifestations are often accompanied by autonomic disturbances and severe abdominal pain, leading to frequent misdiagnosis as primary psychiatric illness [8].

Chronic psychiatric presentations may also occur in several inherited metabolic disorders and are characterized by recurrent mood disturbances, personality changes, anxiety syndromes, and cognitive decline. Such manifestations have been described in Wilson disease, cerebrotendinous xanthomatosis, Niemann–Pick disease type C, and other lysosomal storage diseases [9]. Defects in one-carbon metabolism (methylenetetrahydrofolate reductase (MTHFR) enzyme deficiency and cobalamin metabolic disorders) may impair methylation pathways and contribute to mood dysregulation and neuropsychiatric symptoms [10].

A key contribution of Medjkane and colleagues is showing that psychiatric manifestations in inherited metabolic disorders (IMDs) are not merely late complications [11]. In their case series of 62 children and adolescents, IMD diagnosis occurred at a mean age of ~5.2 years, while the first psychiatric signs appeared at a similar age (~5.6 years), and in many cases, psychiatric symptoms preceded the IMD diagnosis. Overall, 58% displayed psychiatric signs during follow-up, and ~43% of those with psychiatric signs experienced them before the metabolic diagnosis. The authors also described time-linked symptom clusters: (1) signs that tended to occur before or at diagnosis (inattention/impulsivity, disruptive behaviors, sleep problems, and notably aberrant drug response), (2) signs that clustered around diagnosis (including emotional dysregulation and fluctuating symptoms), and (3) later-onset features (dissociative and eating problems). In practice, mood disorders should raise suspicion for IEMs when they are atypical (early onset, episodic confusion, fluctuating course) or multisystemic (neurologic signs, regression) or show unexpected medication response. Medjkane et al. emphasize warning signs such as symptom fluctuation, delirium, isolated visual hallucinations, and poor tolerance/inefficacy of psychotropics—features that, when co-occurring with developmental or somatic clues, should prompt metabolic screening and multidisciplinary assessment.

### 1.2. Unstable Mood May Be the Manifestation of Deficient Methylation Processes

Methylation reactions represent one interface between nutrients and genetic expression. Methylation is the basis of the current interest in S-adenosyl-L-methionine (SAMe) in the treatment of depression [12], as SAMe is a primary methyl donor involved in the synthesis of many neurotransmitters. SAMe is also involved in many methylation reactions in the central nervous system, including the methylation of proteins, nucleic acids, phospholipids, and neurotransmitters.

Mood regulation is tightly coupled to one-carbon metabolism—the biochemical network that supplies methyl groups for DNA and histone methylation (epigenetic regulation), phospholipid and myelin maintenance, and synthesis and catabolism of monoamine neurotransmitters. In this system, dietary folate (as 5-methyltetrahydrofolate (5-MTHF)), vitamin B12, and vitamin B6 help recycle homocysteine to methionine, generating SAMe, the body’s primary methyl donor. When methylation capacity is constrained, through low folate/B12 status, reduced enzyme function (e.g., MTHFR variants), inflammation, oxidative stress, alcohol use, or high metabolic demand, homocysteine can rise, and SAMe availability can fall, creating a low methylation status that plausibly amplifies emotional volatility via neurotransmitter and epigenetic pathways [13].

Low folate status has been associated with poorer antidepressant response in major depression [14]. Beyond unipolar depression, bipolar disorder has also been linked to one-carbon disruption: a meta-analysis reported elevated homocysteine in bipolar disorder during mania and even euthymia, suggesting both state and trait relevance [15].

Genetic evidence further supports this biological link. Meta-analytic work has reported associations between common MTHFR variants (particularly C677T) and major psychiatric disorders, including bipolar disorder and unipolar depression [16,17]. Reduced MTHFR activity limits the formation of bioactive 5-MTHF, potentially impairing methylation capacity.

These findings do not imply that methylation deficiency alone causes mood disorders. Mood disorders are multifactorial conditions. However, the convergence of biomarker, genetic, and treatment-augmentation data suggests that methylation biology may represent a vulnerable pathway in a subset of individuals.

Methylation plays a central role in the epigenetic regulation of stress-response and neuroplasticity pathways, with altered DNA methylation patterns being associated with an increased risk of depression [18].

Similarly, methylation changes in the oxytocin receptor gene (OXTR) have been associated with social anxiety phenotypes [19]. While a causal relationship cannot be established, these findings support the idea that impaired methylation may contribute to emotional dysregulation through gene-regulatory mechanisms. If mood instability in some individuals reflects suboptimal one-carbon metabolism, then nutritional strategies targeting anxiety and depression may be viewed as approaches that enhance methyl donor availability and promote metabolic resilience.

Chronic low-grade inflammation, frequently observed in depression and anxiety disorders, increases oxidative stress and depletes methyl donors. Inflammation also alters neurotransmitter metabolism via activation of the kynurenine pathway, diverting tryptophan away from serotonin production. This creates a functional convergence between inflammatory signaling and methylation stress. Notably, adjunctive L-methylfolate appears particularly effective in depressed individuals with elevated inflammatory markers [20], suggesting that methylation support may partially buffer inflammatory burden [21,22,23]. Dietary anti-inflammatory patterns (Mediterranean-style diet, omega-3 intake, high polyphenol consumption), therefore, may support methylation stability.

### 1.3. Unstable Mood May Be the Result of the Alteration in Gene Expression by Nutrient Deficiency

Nutrients provide energy or structural substrates but also help regulate which genes are turned on or off. It is now well established that nutritional exposures can modify gene expression through epigenetic mechanisms such as DNA methylation, histone modification, and non-coding RNA signaling [24]. These processes are especially relevant during fetal life, infancy, and childhood, when the brain is highly plastic and environmental inputs can leave durable biological marks [25]. Thus, nutrient deficiency may influence mood not only by acutely limiting neurotransmitter synthesis but also by reshaping transcriptional programs involved in neurodevelopment, synaptic plasticity, and stress responsivity [26].

This concept extends beyond classical one-carbon metabolism. Methyl-donor micronutrients such as folate, choline, methionine, betaine, and vitamins B6 and B12 are particularly important because they provide substrates for the epigenetic machinery, but other nutrients are also relevant [27,28,29]. Contemporary nutritional epigenetics suggests that inadequate intake of key micronutrients and essential fatty acids can alter the expression of genes involved in the hypothalamic–pituitary–adrenal axis, inflammatory regulation, dopamine signaling, and neurotrophic pathways [28]. In this framework, a nutritional deficit may bias the expression of stress-related and neuroplasticity-related genes in a direction that increases vulnerability to anxiety and depression [2].

Experimental data provide biologically plausible examples of this mechanism. Early-life iron deficiency provides one of the clearest examples of how nutrient deficiency during development can durably alter gene expression. Reviews of developmental observational studies indicate that iron deficiency during fetal and neonatal life can reprogram gene regulation in the hippocampus, with effects that persist even after later iron repletion [30]. These altered transcriptional profiles involve pathways related to neuronal energy metabolism, dendritic maturation, and epigenetic regulation—changes that are highly relevant because the hippocampus is central to memory, emotional regulation, and stress adaptation [31,32]. In primary experimental work, iron deficiency impaired developing hippocampal neuron gene expression, energy metabolism, and dendrite complexity, supporting the view that nutrient insufficiency during critical windows can produce lasting molecular changes rather than only transient biochemical disturbances [33].

Human data are increasingly consistent with this developmental model. In the EDEN (Etude des déterminants pré- et postnatals précoces du développement et de la santé de l’enfant) mother–child cohort, low maternal adherence to a healthy prenatal dietary pattern was associated with a higher trajectory of anxiety and depression symptoms in offspring from 3 to 8 years of age [34]. Likewise, a large Norwegian prospective cohort found that maternal and early postnatal diet quality were independently related to children’s behavioral and emotional problems by age 5 [35]. These findings do not prove direct causality at the level of gene transcription, but they strengthen the hypothesis that nutrient-dependent biological programming during sensitive periods may influence later emotional stability.

These observations do not mean that mood disorders are genetically “determined” by diet alone nor that one nutrient deficiency explains all cases of depression or anxiety [36]. Mood disorders remain multifactorial. However, the convergence of evidence from epigenetic theory, developmental neuroscience, animal studies, and prospective human cohorts supports the idea that nutrient deficiency can alter gene expression in ways that increase later vulnerability to unstable mood [28]. From this perspective, anti-anxiety and anti-depression nutrition may partly act by preserving a more favorable pattern of gene regulation during critical windows of brain development and adaptation [2,28].

### 1.4. Mood Disorders May Be Long-Latency Deficiency Diseases

Much of classical nutrition was built on the concept of short-latency deficiency diseases, in which a lack of one nutrient produces a relatively specific syndrome within a short time frame [37]. Heaney proposed a broader framework, arguing that many chronic disorders may represent long-latency deficiency diseases: conditions in which suboptimal nutrition exerts pathogenic effects slowly, often over years or decades, before clinical disease becomes apparent [38]. This concept is highly relevant to psychiatry, where the biological foundations of mood disorders may be laid long before symptoms emerge [36].

Applied to depression and anxiety, the long-latency perspective suggests that chronic nutritional inadequacy may gradually alter brain development, stress-system calibration, inflammatory tone, and neuroplastic capacity, eventually manifesting as emotional symptoms much later in life [39]. This possibility is supported by a recent systematic review concluding that early-life undernutrition and later depression are linked across human and animal studies, reinforcing the idea that vulnerability to depression may begin in intrauterine life and continue across childhood and adolescence [40].

Famine studies provide some of the strongest naturalistic evidence for this framework. A systematic review and meta-analysis found that prenatal famine exposure was associated with increased risk of mental disorders, including depression, and with persistent DNA methylation changes in genes involved in neuronal, neuroendocrine, and immune processes [41]. In the Dutch famine cohort, maternal famine exposure before conception was associated with poorer self-reported mental health and higher depressive symptom scores in adult offspring decades later [42]. In the China Health and Retirement Longitudinal Study, severe early-life famine exposure was associated with greater likelihood of depressive symptoms in late adulthood. The strongest associations were observed for exposure during early gestation, especially the first and second trimesters [43].

These observations fit the idea that the psychiatric consequences of nutrient deprivation may be delayed, cumulative, and developmentally programmed rather than immediate. The nutritional insult may occur early, while the clinical phenotype appears only after years of additional developmental demands, psychosocial stress, and biological wear [38,40]. In this sense, at least a subset of mood disorders may be understood as delayed manifestations of long-standing nutritional vulnerability [44]. This perspective does not reduce mood disorders to a single causal pathway. Rather, it highlights that prolonged suboptimal nutrition may become one upstream contributor to later psychiatric illness, especially when exposure occurs during sensitive developmental periods [2,45].

Seen in this light, the clinical relevance of nutrition extends beyond symptomatic adjunctive treatment. Nutritional care may also be preventive, aiming to reduce the accumulation of latent biological risk across the life course [46]. A mood disorder that presents in adolescence or adulthood may, in some individuals, represent the endpoint of a much longer process in which early nutritional inadequacy, developmental programming, and later stress exposures interact over time [41]. This interpretation is particularly important for public health because it places maternal nutrition, childhood diet quality, and early correction of deficiency in the same conceptual space as long-term mental health prevention [47,48].

From a prevention perspective, this concept supports a life-course view of nutrition and mental health. In primary prevention, sustained diet quality during preconception, pregnancy, childhood, and adolescence may help reduce the gradual accumulation of biological vulnerability to later mood disorders [34,35,40,42]. In clinical settings, nutritional strategies are best understood not as stand-alone treatments but as adjunctive approaches that may support standard psychiatric and psychological care over time [35,49]. Their effects are unlikely to be immediate and may require sustained implementation over weeks to months, or longer, depending on baseline nutritional status, inflammatory burden, and the biological pathway being targeted [49,50].

This review examines three interrelated domains: (1) long-chain *n*-3 PUFAs (EPA and DHA) and their effects on neuroinflammation and membrane physiology; (2) the microbiota–gut–brain axis, including probiotic and prebiotic interventions; and (3) micronutrients (vitamins and minerals) that influence neurotransmitter synthesis and immune–metabolic pathways. The aim is to provide a physiologically grounded, food-based framework for anti-anxiety and anti-depression dietary strategies that aligns with the current evidence base. This narrative review is intended for clinicians and translational researchers working at the diet–mental health interface, particularly psychiatrists, nutritionists/dietitians, clinical psychologists, and physiologists.

## 2. Materials and Methods

A targeted literature review was performed using PubMed, Scopus and Web of Science to identify relevant evidence on omega-3 fatty acids, microbiota–gut–brain pathways, and micronutrients involved in neurotransmission and inflammation. Studies were included if they: (1) reported clinical outcomes in depression or anxiety; (2) included dose–response, subgroup, or biomarker-based analyses; and (3) were published in peer-reviewed journals indexed in major bibliographic databases.

Priority was given to systematic reviews, meta-analyses, randomized controlled trials, and representative clinical studies. Experimental studies were included selectively when they provided physiologically relevant insight into neuroinflammatory, membrane, microbial, and immune–metabolic mechanisms related to mood regulation. Reference lists of key articles were additionally screened for relevant sources.

As the purpose of this review was to develop a physiologically grounded, food-based framework rather than to conduct a formal systematic review, the evidence was synthesized narratively, with emphasis on clinically and translationally relevant findings.

## 3. Results

### 3.1. Omega-3 Fatty Acids and Mood/Anxiety Outcomes

Analysis of the literature shows that long-chain *n*-3 PUFAs contribute to neuronal membrane composition and lipid signaling networks that regulate inflammation. Meta-analytic evidence indicates that omega-3 supplementation is associated with reductions in anxiety symptom severity, with larger effects observed in clinical than in non-clinical populations [50].

Evidence from randomized controlled trials suggests that *n*-3 PUFAs, particularly formulations enriched in eicosapentaenoic acid (EPA), may provide modest therapeutic benefit in individuals with depressive disorders. Meta-analyses and meta-regression studies indicate that treatment effects are heterogeneous and influenced by factors such as EPA content, baseline depression severity, and study population characteristics. Notably, dose–response analyses suggest a non-linear association between *n*-3 PUFA dose and improvement in depressive symptoms, with moderate doses appearing more effective than very low or very high intake levels [51,52,53].

A systematic review and meta-analysis pooling 19 clinical trials found that omega-3 treatment was associated with improvement in anxiety symptoms compared with controls, with stronger effects observed in participants with clinical conditions than in subclinical samples [54]. A more recent dose–response meta-analysis suggested that omega-3 supplementation may reduce anxiety symptoms, with the greatest improvements observed at around 2 g/day, although the certainty of evidence was very low [55].

For depression, the evidence base is large but heterogeneous. A Cochrane review of omega-3 supplementation in adults concluded that omega-3s may have a small-to-modest effect on depressive symptoms versus placebo, but this effect was likely not clinically meaningful, and the certainty of the evidence was low to very low [56]. This mixed picture is also reflected in umbrella-level evidence syntheses, which report that findings across meta-analyses are often inconsistent and that the overall strength and credibility of the evidence are generally weak, likely due in part to differences in formulation, dose, study populations, and study quality or risk of bias [57].

One of the most consistent findings is that EPA-predominant formulations tend to perform better than DHA-predominant approaches [58]. Other analyses reported that efficacy was more apparent when EPA constituted the majority of the omega-3 intervention and was delivered at commonly studied doses (often in the 1–2 g/day range), either as monotherapy or as adjunctive treatment [52,53]. In contrast, evidence from prevention-focused randomized trials conducted in generally healthy populations indicates that *n*-3 PUFAs supplementation has little or no effect on preventing the onset of depression or anxiety symptoms, suggesting that potential benefits may be limited primarily to therapeutic rather than preventive contexts [50,51].

### 3.2. Micronutrients, Neurotransmitter Synthesis, and Neuroimmune Regulation

Micronutrients influence mood biology primarily as enzymatic cofactors and signaling molecules that support neurotransmitter synthesis, mitochondrial energy metabolism, antioxidant defense, and immune regulation [59]. In contrast to omega-3 fatty acids, where the evidence base is dominated by broad meta-analytic treatment effects, micronutrient data are more nutrient-specific and often more dependent on baseline status, inflammatory burden, or biomarker-defined subgroups [60,61]. Overall, the literature suggests that micronutrient interventions are most clinically relevant when they correct insufficiency or are used adjunctively in selected patients rather than as universal treatments for depression or anxiety [49,62].

Folate and vitamin B12 are central to one-carbon metabolism and methylation capacity, which in turn influence monoamine synthesis, phospholipid metabolism, and treatment response [63]. In clinical studies, adjunctive L-methylfolate has shown benefit in SSRI-resistant depression, with stronger signals in biomarker- and genotype-defined subgroups, supporting the view that folate-related interventions may be most useful in patients with impaired methylation capacity or treatment resistance rather than in unselected populations [21]. Vitamin B6 also deserves attention because it serves as a cofactor for amino acid decarboxylation reactions and for glutamate decarboxylase, thereby linking B6 status to γ-aminobutyric acid (GABA) synthesis and inhibitory–excitatory balance [64]. A randomized trial in young adults found that high-dose vitamin B6 reduced self-reported anxiety and altered sensory processing in a way consistent with strengthened inhibitory function [65].

Magnesium and zinc are among the most frequently studied minerals in nutritional psychiatry. Magnesium is relevant to neuronal excitability through effects on NMDA receptor signaling and broader neurophysiologic stability [66]. A randomized clinical trial reported improvement in mild-to-moderate depressive symptoms with magnesium supplementation, and more recent meta-analytic synthesis suggests a potentially beneficial effect overall, although trial quality and consistency remain variable [67]. Zinc, meanwhile, participates in synaptic signaling, neuroplasticity, and immune regulation. Meta-analytic evidence indicates that lower peripheral zinc levels are associated with depression, while supplementation studies suggest that zinc may improve depressive symptoms in some settings, particularly in older adults, individuals with mild-to-moderate depressive symptoms, overweight or obese patients with depressive symptoms, and certain clinically defined subgroups such as antidepressant-resistant patients [68,69]. Importantly, the clinical relevance of dietary zinc and magnesium depends not only on intake but also on bioavailability, which may vary according to the food matrix; for example, phytate-rich foods can reduce mineral absorption [70,71,72].

Vitamin D has pleiotropic actions relevant to mood, including immune modulation, neurotrophic signaling, and possible effects on neurotransmission [73]. Meta-analyses of randomized trials report mixed but generally small reductions in depressive symptoms, with more favorable results in some clinically depressed, short-term, or deficiency-defined subgroups; however, evidence for anxiety outcomes is weaker and often non-significant [74,75]. This pattern suggests that vitamin D is better viewed as a targeted correction strategy than as a broadly effective anti-anxiety or antidepressant intervention in the general population.

Vitamin C and copper are less often discussed in psychiatric nutrition trials, yet their biochemical relevance is strong. Vitamin C is highly concentrated in the brain, where it contributes to redox balance and catecholamine biology [76]. Copper handling is likewise important because dopamine beta-hydroxylase, the enzyme that converts dopamine to norepinephrine, is copper-dependent and uses ascorbate as a cofactor [77]. In parallel, catecholamine synthesis remains constrained upstream by tyrosine hydroxylase, the rate-limiting enzyme in dopamine production [78]. These pathways reinforce a general principle seen across the micronutrient literature: neurotransmitter synthesis depends not only on precursor availability but also on adequate cofactor status and a metabolic environment not dominated by inflammation or oxidative stress [79].

### 3.3. The Microbiota–Gut–Brain Axis and Psychobiotic Interventions

Most published reviews describe the microbiota–gut–brain axis as an integrated network encompassing vagal signaling, immune activation, microbial metabolites (including short-chain fatty acids), and neuroendocrine stress responses [80,81]. Stress and depressed mood can remodel microbial communities, while microbiome-derived products can influence neuroinflammation, neurotransmitter precursors, and behavior [82,83]. Clinical trials of probiotic and prebiotic interventions in major depressive disorder have produced variable results. Some randomized trials report improvements in depressive symptoms and metabolic or inflammatory markers following probiotic administration, while others show minimal effects, highlighting heterogeneity in strains, dosing, baseline diet, and clinical severity [84,85,86,87].

Recent meta-analyses suggest small overall benefits of probiotics and prebiotics for depressive symptoms, with strain-specific effects and substantial heterogeneity across studies. These findings support a cautious interpretation and favor phenotype- and product-specific applications rather than broad claims about “probiotics” as a homogeneous category [81,83,88].

## 4. Discussion

*n*-3 PUFAs (EPA and DHA) have become a “hot topic” in many scientific discussions because they sit at the intersection of neuroinflammation, membrane biology, and stress physiology. Emerging evidence suggests that these mechanisms may partly explain the associations between dietary fatty acid intake and psychiatric outcomes [89,90]. Modern dietary patterns often skew toward higher omega-6 intake and lower omega-3 intake, a shift that may matter for emotional regulation, given that EPA/DHA influence inflammatory mediators, neurotransmission, and neuronal membrane properties [89,90,91,92]. Clinically, the question is not whether omega-3s are good for the brain in general but whether increasing EPA/DHA meaningfully improves depressive and anxiety symptoms and in which patients.

EPA is often considered more relevant for modulating inflammatory pathways, whereas DHA is more structurally concentrated in neuronal membranes [93]. In mood disorders, EPA-predominant formulations may therefore be particularly beneficial in individuals with co-occurring metabolic dysfunction or low-grade inflammation [94,95].

From an epigenetic perspective, PUFAs can interact with one-carbon metabolism and DNA methylation processes, which regulate gene expression without altering the DNA sequence. One-carbon metabolism depends on nutrients such as folate, vitamin B12, methionine, and choline to generate S-adenosylmethionine (SAM), the universal methyl donor used in DNA and histone methylation reactions. Experimental studies indicate that fatty acids can influence the activity of DNA methyltransferases (DNMTs) and thereby modify methylation patterns of genes involved in metabolic and inflammatory regulation [96]. Omega-3 fatty acids have been shown to affect the epigenetic regulation of genes involved in immune signaling, lipid metabolism, and neuronal function, suggesting a potential pathway by which dietary lipids may influence neurobiological processes relevant to mood regulation [97]. Epigenetic alterations have been reported in several genes implicated in depression, including those involved in neurotrophic signaling, stress-response pathways, and inflammatory regulation, highlighting the relevance of methylation processes in psychiatric disorders [96].

In parallel, omega-3 PUFAs exert anti-inflammatory effects, which are increasingly recognized as important in the pathophysiology of depression and related mood disorders. EPA and DHA modulate inflammatory responses by competing with arachidonic acid (an omega-6 fatty acid) for enzymatic pathways involved in eicosanoid synthesis. This competition results in decreased production of pro-inflammatory mediators such as prostaglandins and leukotrienes while promoting the formation of specialized mediators, including resolvins and protectins [89,98]. These lipid mediators contribute to the resolution of inflammation and may influence neuroimmune signaling within the central nervous system. More specifically, omega-3-derived specialized pro-resolving mediators (SPMs), including resolvins and protectins, should be viewed not merely as anti-inflammatory metabolites but as endogenous signals that actively orchestrate the resolution phase of inflammation. Rather than only reducing inflammatory mediator production, they help terminate inflammatory responses by promoting return to tissue homeostasis and facilitating resolution pathways in immune and brain-relevant cellular systems. In microglial models, resolvin D1 and resolvin E1 have been shown to promote resolution of inflammation, and in patients with major depressive disorder clinical response to EPA supplementation has been associated with higher plasma concentrations of pro-resolving lipid mediators [99,100].

Clinical and experimental studies have also shown that omega-3 supplementation can reduce circulating levels of inflammatory markers, including interleukin-6 (IL-6), tumor necrosis factor-α (TNF-α), and C-reactive protein (CRP), which have been associated with depressive symptomatology [90].

The epigenetic and anti-inflammatory actions of PUFAs are likely interconnected. Inflammatory signaling can itself modify DNA methylation patterns, while epigenetic regulation can influence the transcription of inflammatory genes. By simultaneously modulating methylation pathways and immune signaling, omega-3 fatty acids may affect multiple biological systems involved in mood disorders [89,97]. These mechanisms may also help explain why omega-3 supplementation appears to produce stronger clinical effects in individuals with elevated inflammatory markers or clinically defined depression, compared with low-risk populations.

Omega-3s appear more reliable as a treatment adjunct than as a universal prevention strategy (Table 1). A meta-analysis focused on the prevention of depression and anxiety found limited evidence for preventive benefit across general populations, suggesting that PUFAs may not act as a broad prophylactic in low-risk groups [50]. The distinction between treatment and prevention aligns with a targeted nutritional intervention: omega-3 supplementation may yield clearer benefits when baseline symptom severity is higher, clinical status is well-defined, or when modifiable biological processes such as inflammation are present [56].

Micronutrient findings support a more conditional and biologically targeted interpretation than the omega-3 literature (Table 2). Rather than functioning as universal antidepressant or anxiolytic agents, vitamins and minerals appear most relevant when they correct latent insufficiency or support pathways already constrained by metabolic stress, inflammation, or impaired methylation [101,102]. Folate, vitamin B12, and vitamin B6 are particularly important because they influence one-carbon metabolism, monoamine turnover, and GABA synthesis, while magnesium and zinc are linked to neuronal excitability, synaptic plasticity, and immune signaling [103,104,105]. Vitamin D appears to follow a similar pattern, with modest average effects but more favorable signals in clinically depressed participants or in subgroups with documented nutrient deficiency at baseline [106]. The biochemical relevance of vitamin C and copper further reinforces that catecholamine synthesis depends not only on precursor availability but also on cofactor sufficiency and redox balance [107]. Taken together, these data suggest that micronutrients should be viewed less as stand-alone psychotropic interventions and more as permissive factors that determine whether neurotransmitter synthesis, mitochondrial function, and neuroimmune regulation can proceed efficiently. This may explain why targeted correction of inadequacy is more consistently beneficial than indiscriminate supplementation, and why micronutrient density at the level of the whole dietary pattern is likely more clinically relevant than any single nutrient in isolation [108]. For some nutrients, particularly vitamin D and folate, the relationship with health is unlikely to be simply linear, as both deficiency and excessive supplementation may be undesirable; this further supports a targeted approach based on nutritional status rather than routine high-dose use [109,110]. Table 2 provides an overview of selected micronutrients implicated in neurotransmitter synthesis and neuroimmune regulation, together with their principal biochemical functions, relevance to depression and anxiety, and representative food sources.

The microbiota–gut–brain axis represents a complex, bidirectional communication network integrating neural, immune, endocrine, and metabolic signaling pathways. Disruptions in this system—often conceptualized as dysbiosis—have been increasingly associated with altered emotional regulation and psychiatric symptoms [111].

A central theme across the literature is the broad diversity through which gut microbiota influence brain function. These include modulation of immune responses, production of microbial metabolites, and direct neural signaling via the vagal nerve. Microbiota-derived metabolites, such as short-chain fatty acids (SCFAs), play a particularly important role by regulating intestinal barrier integrity, systemic inflammation, and even CNS processes. SCFAs, especially butyrate, exhibit anti-inflammatory and neuroactive properties, and their depletion—often linked to low dietary fiber intake—may contribute to increased gut permeability and endotoxemia, both implicated in depression pathophysiology [112].

In addition to metabolic signaling, the gut microbiota influences neurotransmitter systems. Notably, microbial regulation of the tryptophan–kynurenine pathway can alter serotonin availability, a key factor in mood regulation [113]. However, current understanding suggests that depression cannot be fully explained by monoamine deficiency alone, with neuroplasticity, neurogenesis, and inflammatory pathways playing equally important roles [114]. Experimental evidence demonstrates that depressive-like behaviors can be transferred via fecal microbiota transplantation, supporting a causal role of microbial composition in mood regulation [115].

One of the most robust biological links between gut microbiota and mood disorders is chronic low-grade inflammation. Alterations in microbiota composition can impair intestinal barrier integrity, leading to increased permeability (“leaky gut”) and translocation of bacterial components such as lipopolysaccharides into systemic circulation. This triggers immune activation and pro-inflammatory cytokine release [116,117,118].

Reduced abundance of butyrate-producing bacteria compromises epithelial barrier function and facilitates endotoxemia, a process strongly associated with depression-related inflammation. Systemic inflammation can subsequently influence brain function by increasing cytokine penetration across the blood–brain barrier, activating microglia, and altering neurotransmitter metabolism [118]. Probiotics may counteract this pathway by enhancing gut barrier integrity, reducing LPS translocation, and by downregulating pro-inflammatory cytokines [119]. This positions inflammation modulation as a central mechanism of psychobiotic action.

Gut microbes metabolize dietary substrates into bioactive compounds, particularly SCFAs such as acetate, propionate, and butyrate. These metabolites represent a key biochemical interface between microbiota and host physiology. SCFAs serve as energy sources for colonocytes, strengthen gut barrier integrity, exhibit anti-inflammatory and immunomodulatory properties, and influence central nervous system functioning [119,120]. Butyrate is particularly important because it acts as a histone deacetylase inhibitor, influencing gene expression, modulates neuroplasticity and brain-derived neurotrophic factor (BDNF) and affects blood–brain barrier integrity [83]. Reduced SCFA production, often due to low fiber intake or dysbiosis, can therefore contribute to neuroinflammation and impaired neuroplasticity, both hallmarks of depression.

The gut microbiota plays a significant role in regulating neurotransmitter systems, particularly serotonin, GABA, and dopamine. A critical pathway in this context is the tryptophan–kynurenine axis, whereby tryptophan can be metabolized into serotonin or diverted into kynurenine metabolites, some of which are associated with neurotoxicity and depression [113,121]. The gut microbiota influences this balance through immune activation and metabolic signaling pathways. Additionally, certain *Lactobacillus* and *Bifidobacterium* strains are capable of producing GABA, while microbial metabolites can also modulate dopaminergic signaling [122]. Probiotics may therefore shift tryptophan metabolism toward serotonin production, enhance inhibitory neurotransmission (via GABA), and improve emotional regulation.

The HPA axis is a central stress-response system frequently dysregulated in depression and anxiety. The gut microbiota can influence HPA axis activity through multiple pathways, including immune signaling (e.g., cytokine-mediated effects on hypothalamic function), neural mechanisms such as vagal stimulation, and microbial metabolite signaling. Experimental studies have demonstrated that germ-free animals exhibit exaggerated stress responses, whereas restoration of the microbiota can normalize HPA axis activity [112,123,124]. Probiotics may attenuate stress responses by reducing cortisol levels, improving resilience to psychological stress, and stabilizing HPA axis feedback loop.

Another relevant mechanism is the regulation of BDNF, a key protein involved in synaptic plasticity, learning and memory, and antidepressant response. The gut microbiota may influence BDNF levels indirectly via SCFAs through epigenetic, as well as inflammatory pathways and modulation of neurotransmitter systems. Furthermore, microbiota-targeted interventions, including flavonoid-rich dietary patterns that alter gut microbial composition, have been associated with increased BDNF levels and improvements in depressive symptoms [125,126].

Probiotics, as a major class of psychobiotics, show promising but inconsistent effects in clinical studies. Their efficacy is limited by substantial heterogeneity in study design, strains, dosages, and populations, as well as the predominance of studies in healthy or mildly symptomatic individuals, which restricts clinical applicability. Moreover, the link between microbiome changes and improvements in mood remains poorly defined. Table 3 provides an overview of psychobiotic and probiotic mechanisms relevant to mood regulation, highlighting their biochemical roles, implications for depression and anxiety, and representative food sources or targeted interventions.

Prebiotics and synbiotics also demonstrate therapeutic potential, primarily through their ability to selectively stimulate beneficial microbial populations. For example, fructooligosaccharides and galactooligosaccharides have been associated with improvements in anxiety and depressive symptoms, alongside increases in *Bifidobacterium* abundance. These findings support the concept that targeting overall microbial ecology, rather than individual strains alone, may represent a more effective therapeutic strategy [112,118].

An important consideration emerging from recent literature is the role of individual variability in microbiota composition. The baseline microbial profile appears to influence responsiveness to dietary and probiotic interventions, suggesting that personalized or precision approaches may be necessary for optimal outcomes. This aligns with broader trends in precision nutrition and psychiatry, where interventions are tailored based on biological, environmental, and lifestyle factors [127,128].

While preclinical data strongly support a role for the gut microbiota in mood regulation, clinical evidence remains in its early stages. Larger, well-controlled trials in clinically diagnosed populations are needed, alongside standardized methodologies for microbiome assessment. Additionally, integrating multi-omics approaches (e.g., metabolomics, transcriptomics) could provide deeper insights into functional microbial changes and their relationship with neuropsychiatric outcomes.

### 4.1. Why Dietary Patterns Outperform Single Nutrients

**Figure 1:**
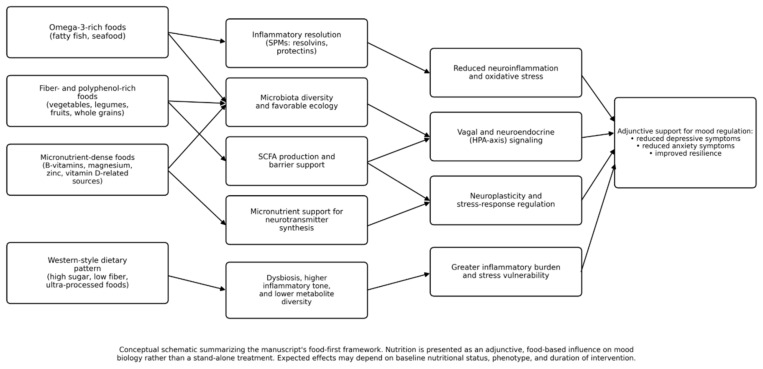
Conceptual food-first framework linking diet quality to depression- and anxiety-related biological pathways.

Inflammation is a plausible common amplifier across depression and anxiety phenotypes. Pro-inflammatory cytokines can influence neurotransmitter turnover, glutamatergic signaling, and neuroplasticity, and can shift tryptophan metabolism away from serotonin production (Figure 1). Omega-3 fatty acids may mitigate inflammatory signaling through altered lipid mediator profiles, while fiber-rich dietary patterns can reduce endotoxin exposure and support SCFA-mediated immune regulation. These converging pathways help explain why single-nutrient effects are often modest and why broader dietary patterns may yield more robust clinical signals [51,54,81,129].

Diet emerges as a primary modulator of gut microbiota composition and function, thereby indirectly influencing mental health. Diets rich in fiber, polyphenols, and omega-3 fatty acids promote microbial diversity and the growth of beneficial taxa such as *Bifidobacterium* and *Lactobacillus*, which are frequently associated with improved psychological outcomes. Conversely, Western-style diets characterized by high fat and sugar intake are linked to reduced microbial diversity and increased abundance of pro-inflammatory bacteria, potentially exacerbating mood disorders [130]. The concept of “psychobiotics” has thus emerged to describe dietary or microbial interventions that confer mental health benefits through microbiota-mediated mechanisms [131].

Whole-diet interventions affect multiple systems simultaneously: omega-3 availability, micronutrient density, glycemic stability, and the microbiome. The SMILES trial provides proof of concept that improving diet quality can reduce depressive symptoms over 12 weeks when delivered with behavioral support from a dietitian. The multi-target approach may provide resilience to variations in underlying pathophysiology and could be more practical for long-term use than relying solely on high-dose supplementation [132].

### 4.2. Translational Implications: Defining “Anti-Depression” and “Anti-Anxiety” Foods

If the biological mechanisms are emphasized, foods most likely to support mood and anxiety regulation are those that provide long-chain *n*-3 PUFAs, diverse fermentable fibers and polyphenols that promote favorable microbial metabolite production, and micronutrients required for neurotransmitter synthesis and mitochondrial energy metabolism. In practice, this corresponds to a Mediterranean-style dietary pattern centered on vegetables, legumes, whole grains, nuts, seeds, extra-virgin olive oil, and regular seafood intake. Supplementation is best reserved for documented deficiency states or selected clinical contexts, such as vitamin D deficiency, adjunctive L-methylfolate in SSRI-resistant depression, or individualized use of omega-3 or micronutrient supplementation when baseline diet and symptom profile support it.

### 4.3. Limitations and Research Priorities

The evidence is limited by heterogeneity in diagnostic criteria, baseline nutrient status, interventions, and outcome measures, as well as by short trial durations relative to dietary and neuroplastic changes. Future research should adopt precision-nutrition approaches, incorporating baseline biomarkers (e.g., omega-3 index, hs-CRP, vitamin D, micronutrient status, microbiome profiles) and stratified designs. Standardized reporting of dosage, adherence, and concurrent diet would further improve interpretability.

### 4.4. Future of Personalized Nutrition

Looking ahead, nutritional psychiatry is likely to move toward more personalized approaches. Metabolomics, biomarker profiling, and microbiome sequencing may help define biologically meaningful subgroups and identify individuals most likely to benefit from targeted dietary strategies or selective supplementation. In this context, future progress will depend on integrating dietary assessment with molecular and microbial phenotyping while ensuring that such precision-based approaches are validated in robust clinical trials before routine implementation.

## 5. Conclusions

Current evidence supports a food-first approach to mood, emphasizing improved diet quality, diverse fiber intake, adequate micronutrient status, and regular consumption of omega-3-rich seafood. Omega-3 supplementation appears to show more consistent benefits for anxiety, whereas effects on depression are more heterogeneous and should be interpreted cautiously; preventive effects in generally healthy populations remain limited. Psychobiotics yield small, strain- and phenotype-dependent benefits, while micronutrient supplementation is the most effective when correcting deficiencies or in treatment-resistant cases. Overall, individualized nutritional assessment and dietary optimization should be prioritized over universal supplementation.

## Figures and Tables

**Table 1:** Omega-3 fatty acids in mood regulation.

Principal Biochemical Role	Mood-Related Relevance	Representative Food Sources
Incorporated into neuronal membrane phospholipids (especially DHA), influencing membrane fluidity and receptor function	Supports synaptic function, neuroplasticity, and cognitive processes relevant to mood regulation	Fatty fish (salmon, mackerel, sardines), seafood
Precursor to anti-inflammatory lipid mediators (especially EPA-derived resolvins and protectins)	Reduces neuroinflammation, which is implicated in depression and anxiety	Fatty fish, fish oil supplements
Modulation of neurotransmitter systems (serotonin, dopamine) and signal transduction pathways	Contributes to improved emotional regulation and stress response	Fatty fish, algae-based omega-3 sources
Regulation of gene expression via nuclear receptors and epigenetic mechanisms	Influences pathways involved in neuroplasticity and resilience to stress	Fatty fish, omega-3-enriched foods
Interaction with the gut microbiota and promotion of beneficial microbial profiles	May indirectly affect mood via the microbiota–gut–brain axis and metabolite production	Fatty fish; indirectly supported by omega-3-rich dietary patterns (e.g., Mediterranean diet)

**Table 2:** Selected micronutrients relevant to neurotransmitter synthesis and neuroimmune regulation in depression and anxiety.

Micronutrient	Principal Biochemical Role	Mood-Related Relevance	Representative Food Sources
Folate (L-methylfolate)	One-carbon metabolism; methyl donor generation; supports monoamine synthesis and methylation reactions	May influence antidepressant response, especially in impaired methylation states	Leafy greens, legumes, asparagus, avocado, citrus fruits
Vitamin B12	Works with folate in methionine/homocysteine cycling and methylation capacity	Deficiency may contribute to low mood, cognitive symptoms, and poorer emotional resilience	Fish, meat, eggs, dairy products, fortified cereals
Vitamin B6	Cofactor for amino acid decarboxylation reactions; required for glutamate decarboxylase	May support inhibitory tone and reduce hyperexcitability linked to anxiety	Poultry, fish, bananas, potatoes, chickpeas, whole grains
Vitamin D	Immune modulation, neurotrophic signaling, possible effects on neurotransmission	More relevant in deficient or clinically vulnerable subgroups than as a universal intervention	Fatty fish, egg yolk, fortified milk, fortified dairy alternatives
Magnesium	Modulates NMDA receptor activity, neuronal excitability, and cellular energy metabolism	May reduce depressive symptoms and support neurophysiologic stability	Nuts, seeds, legumes, spinach, whole grains, dark chocolate
Zinc	Synaptic signaling, neuroplasticity, antioxidant and immune functions	Low zinc status has been associated with depressive states and impaired stress adaptation	Oysters, red meat, pumpkin seeds, legumes, nuts, dairy
Vitamin C	Brain antioxidant; supports catecholamine synthesis; maintains redox homeostasis	Relevant to catecholamine balance and protection against oxidative neurobiological stress	Citrus fruits, kiwi, berries, bell peppers, broccoli
Copper	Cofactor for dopamine beta-hydroxylase and other neural enzymes	Important for catecholamine balance, although both deficiency and dysregulation may be problematic	Shellfish, liver, nuts, seeds, cocoa, whole grains
Tyrosine-dependent catecholamine pathway	Tyrosine hydroxylase is the rate-limiting step in dopamine synthesis	Highlights that neurotransmission depends not only on amino acid supply but also on micronutrient adequacy	Protein-rich foods such as poultry, fish, dairy, soy, legumes

**Table 3:** Psychobiotics and probiotics in mood regulation.

Principal Biochemical Role	Mood-Related Relevance	Representative Food Sources/Interventions
Modulation of the microbiota–gut–brain axis via vagal signaling, immune pathways, and neuroendocrine (HPA axis) regulation	Influences stress response, emotional regulation, and resilience to anxiety and depression	Probiotic supplements (strain-specific); fermented foods (yogurt, kefir, sauerkraut, kimchi)
Production of microbial metabolites, including short-chain fatty acids (SCFAs)	SCFAs support gut barrier integrity, reduce neuroinflammation, and modulate brain function	Fiber-rich diets supporting endogenous microbiota; prebiotics
Regulation of immune signaling and systemic inflammation	Reduction in pro-inflammatory cytokines linked to depressive symptoms	Probiotic and synbiotic formulations; diet patterns supporting microbiome diversity
Modulation of neurotransmitter systems (e.g., GABA, serotonin precursors)	Contributes to improved mood, reduced anxiety, and altered stress reactivity	Specific strains (e.g., *Lactobacillus*, *Bifidobacterium*)
Influence on microbial composition and diversity (strain- and host-dependent effects)	Associated with small, heterogeneous improvements in depressive symptoms; effects depend on baseline microbiota and clinical phenotype	Targeted psychobiotic interventions; personalized probiotic/prebiotic strategies

## Data Availability

No new data were created or analyzed in this study. Data sharing is not applicable to this article.

## Abbreviations

The following abbreviations are used in this manuscript:

BDNF	Brain-Derived Neurotrophic Factor
DHA	Docosahexaenoic Acid
EDEN	Etude des déterminants pré- et postnatals précoces du développement et de la santé de l’enfant
EPA	Eicosapentaenoic Acid
GABA	Gamma-aminobutyric Acid
IEMs	Inborn Errors of Metabolism
IMD	Inherited Metabolic Disorders
MTHF	Methylenetetrahydrofolate
MTHFR	Methylenetetrahydrofolate Reductase
*n*-3 PUFAs	Omega-3 Polyunsaturated Fatty Acids
OXTP	Oxytocin Receptor Gene
RCTs	Randomized Controlled Trials
SAMe	S-adenosyl-L-methionine
SCFAs	Short-Chain Fatty Acids
